# Case Report: A case of diffuse subretinal fibrosis and uveitis syndrome with vitreoretinal traction syndrome

**DOI:** 10.3389/fmed.2026.1671793

**Published:** 2026-05-07

**Authors:** Xiaohua Zhang, Yongping Hu, Jian Li

**Affiliations:** Department of Ophthalmology, Affiliated Hangzhou First People’s Hospital, School of Medicine, Westlake University, Hangzhou, Zhejiang, China

**Keywords:** anti-vegf, macular edema, retinal neovascularization, subretinal fibrosis, subretinal fibrosis and uveitis syndrome, uveitis, vitreoretinal traction syndrome

## Abstract

**Purpose:**

This study aimed to report a case of diffuse subretinal fibrosis and uveitis (SFU) syndrome combined with vitreoretinal traction (VRT) syndrome, with a 4-year interval between the involvement of the two eyes in an elderly woman.

**Case description:**

A 67-year-old woman presented with sudden, painless vision loss in her left eye for 20 days. The right eye had experienced blurred vision for more than 4 years without treatment. Best-corrected visual acuity (BCVA) was 0.02 in the right eye and 0.05 in the left eye. Anterior segment examination was unremarkable, with no signs of anterior inflammation or iris nodules in either eye. In the right eye, clumps of white deposits behind the intraocular lens (IOL) and granular white opacities in the vitreous were observed. Diffuse yellowish-white subretinal lesions of varying sizes were present throughout the fundus, extending from the posterior pole to the far periphery, accompanied by scattered areas of hyperpigmentation and depigmentation. The lesion boundaries were relatively well defined. In the left eye, flocculent vitreous opacities were observed, some of which were adherent to the retina. Hemorrhages and scattered small yellowish-white subretinal lesions were present in the posterior fundus. Retinal degeneration was also noted in the mid-peripheral retina. Comprehensive ophthalmologic and systemic examinations were performed. Fundus fluorescein angiography (FFA) revealed secondary neovascular membranes in the posterior pole of the retina. However, systemic evaluation did not reveal any significant abnormalities. The patient was diagnosed with bilateral SFU syndrome, VRT syndrome, and retinal neovascularization in the left eye. Pars plana vitrectomy and intraocular injection of triamcinolone acetonide were performed. Postoperatively, systemic glucocorticoids and immunosuppressive therapy were administered. After more than 1 year of follow-up, the macular edema recurred repeatedly, while the subretinal fibrosis remained largely stable. Ultimately, this study also attempted anti-vascular endothelial growth factor (VEGF) therapy for macular edema and achieved a partial therapeutic effect.

**Conclusion:**

SFU syndrome is a rare condition. The present case demonstrated a 4-year interval between bilateral onset and was further complicated by VRT syndrome and retinal neovascularization, features that have not been previously reported. Glucocorticoids and immunosuppressive agents may delay or alleviate subretinal fibrosis to some extent; anti-VEGF therapy can provide therapeutic benefit for macular edema in this disease.

## 1 Introduction

Diffuse subretinal fibrosis and uveitis (SFU) syndrome is a rare, chronic, and potentially devastating inflammatory disorder. Characterized by the development of progressive subretinal fibrosis following episodes of choroidal inflammation, SFU is typically characterized by white or yellowish choroidal lesions in the posterior pole that coalesce over weeks to months into extensive fibrotic plaques, often leading to irreversible structural damage and significant vision loss (1–3). While first described in the mid-20th century by Adalbert Fuchs as “choroiditis proliferans,” the condition has since been recognized as a distinct clinical entity with a poorly understood pathogenesis, although an autoimmune-mediated process targeting the retinal pigment epithelium (RPE) and inner choroid is suspected (4, 5).

SFU predominantly affects adults who are otherwise healthy and is typically bilateral, though the onset may be sequential (6). The clinical presentation and natural history of SFU share considerable overlap with other inflammatory chorioretinopathies, most notably multifocal choroiditis (MFC) and punctate inner choroidopathy (PIC). This has led to a prevailing view among some researchers that these conditions may represent a spectrum of the same underlying disease process, rather than entirely separate entities (7, 8). In many cases, the initial inflammatory phase may be subtle or even subclinical, with the diagnosis only becoming apparent once subretinal fibrosis has already compromised macular architecture (2, 9).

A major clinical challenge in managing SFU lies in its poor prognosis. Even when active inflammation is controlled with systemic immunosuppression, the pre-existing subretinal fibrosis remains, and visual recovery is often limited due to irreversible damage to the outer retinal layers and RPE (10). Moreover, the differential diagnosis of SFU is broad and includes infectious etiologies (such as syphilis and tuberculosis) and masquerade syndromes.

Despite the significant morbidity associated with SFU, comprehensive clinical descriptions are sparse, and evidence-based treatment protocols remain poorly defined. Recent literature has highlighted the potential role of biologic agents, such as tumor necrosis factor-alpha (TNF-α) inhibitors and rituximab, in refractory cases, yet their use is often limited by cost and availability (11, 12). The variable clinical course, coupled with a lack of standardized management guidelines, underscores the importance of detailed case reports that document clinical nuances, therapeutic decision-making, and long-term outcomes.

In this context, this study describes a case of a 67-year-old woman with bilateral SFU syndrome, with a 4-year interval between the involvement of the two eyes, complicated by vitreoretinal traction (VRT) syndrome and retinal neovascularization in the left eye. This case highlights the diagnostic challenges in differentiating SFU from masquerade syndromes, the rationale for surgical intervention in the setting of significant VRT, and the long-term management of recurrent macular edema.

## 2 Case description

Written informed consent was obtained from the patient and her son, who agreed to the publication of the patient’s identifiable data, including age, race, gender, imaging data, hematological examinations, and medical history. The study protocol was approved by the Institutional Review Board of Hangzhou First People’s Hospital and was conducted in accordance with the Declaration of Helsinki.

A 67-year-old woman presented with a 20-day history of painless vision loss in the left eye. The patient experienced a similar sudden vision loss in the right eye 4 years ago, but did not pay attention to it and did not seek medical attention or treatment. She was highly myopic in both eyes and had undergone cataract surgery and pterygium excision more than 10 years previously. There was no recent history of influenza or fever. She also denied any history of infection or hereditary disease.

The best-corrected visual acuity (BCVA) was 0.02 in the right eye and 0.05 in the left eye. Intraocular pressure was within the normal range in both eyes. Anterior segment examination was unremarkable, with no evidence of anterior inflammation or iris nodules in either eye.

**Figure 1:**
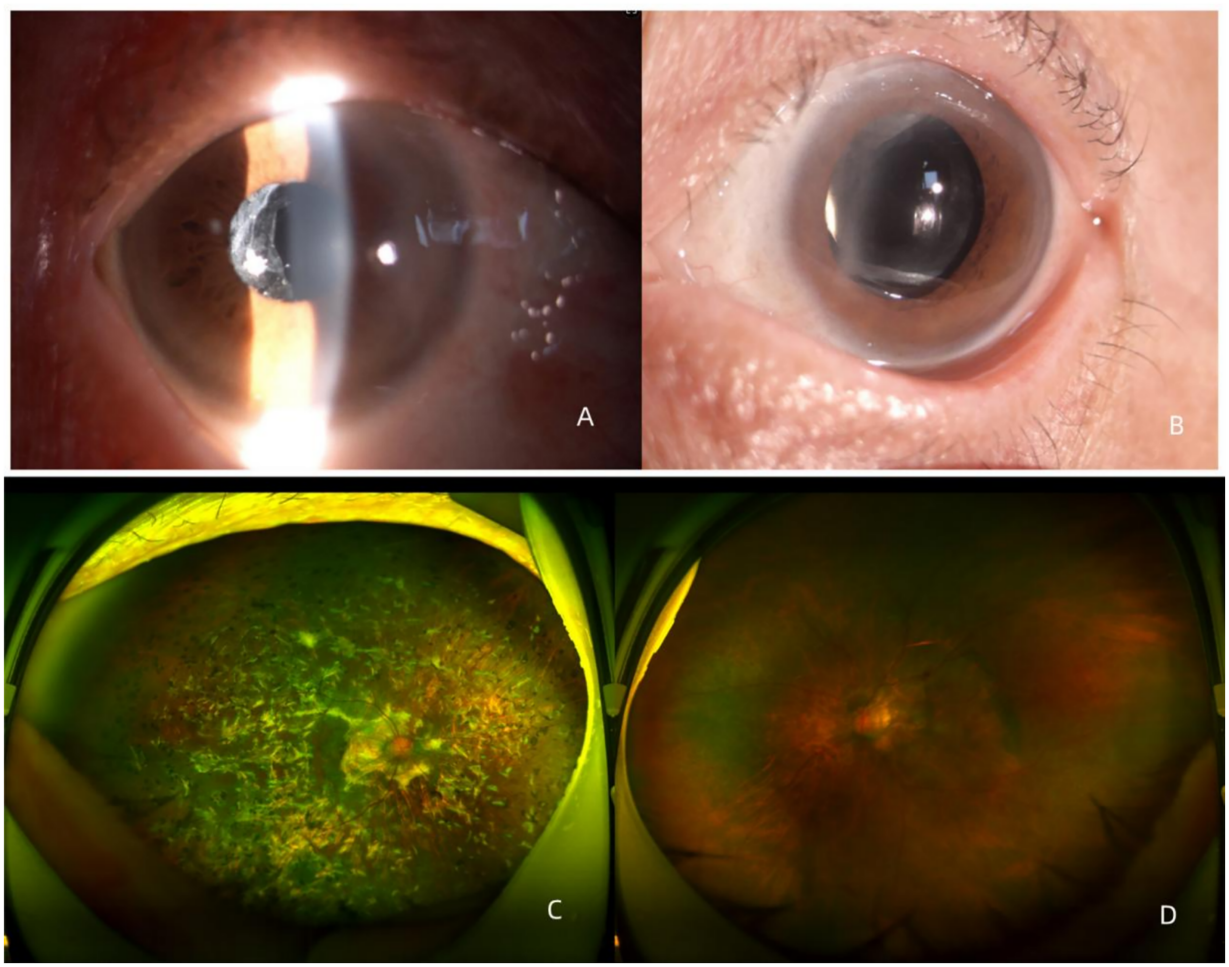
Binocular anterior segment and fundus photography. **(A)** Anterior segment photograph of the right eye; **(B)** Anterior segment photograph of the left eye; **(C)** Fundus photograph of the right eye; and **(D)** Fundus photograph of the left eye.

In the right eye, clumps of white deposits were observed behind the intraocular lens (IOL), along with granular white vitreous opacities (Figure 1A). Diffuse yellowish-white subretinal lesions of varying sizes were present throughout the fundus, extending from the posterior pole to the far periphery and accompanied by scattered hyperpigmented and depigmented changes. The lesion boundaries were relatively clear (Figure 1C).

In the left eye, the anterior segment was normal, and the IOL was in place (Figure 1B). Flocculent vitreous opacities were observed, some of which were adherent to the retina. Hemorrhage and scattered small yellowish-white subretinal lesions were presented in the posterior fundus. Retinal degeneration was also observed in the mid-peripheral retina (Figure 1D).

**Figure 2:**
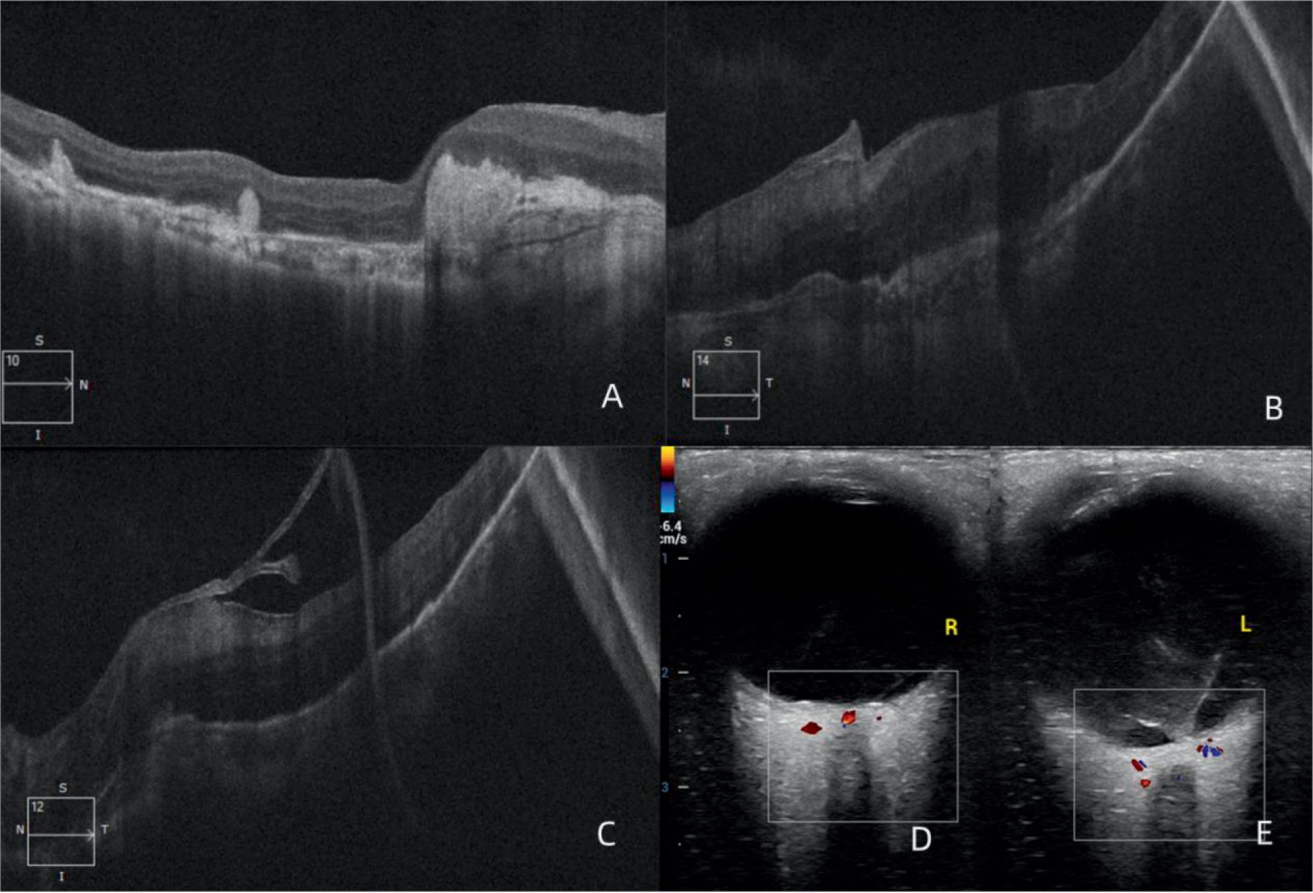
Binocular optical coherence tomography (OCT) and ocular B-ultrasound findings. **(A,B)** Macular OCT examination of the right and left eye, respectively; **(C)** OCT of the left eye shows the presence of vitreoretinal traction; and **(D,E)** ocular ultrasonography of the right and left eye, respectively.

Optical coherence tomography (OCT) B-scan demonstrated extensive subretinal fibrosis in the right eye, without evidence of neovascular membrane formation or exudation, and with marked loss of the outer retinal layers (Figure 2A). In the left eye, planar hyperreflective subfoveal material associated with intraretinal cystoid edema was observed (Figure 2B). In addition, highly reflective membranous bands were visible in the vitreous cavity, connected to the retinal surface (Figure 2C).

Ocular ultrasonography revealed thickening of the posterior wall of the right eye near the optic disk, with slightly increased internal echoes (Figure 2D). In the left eye, punctate and linear echoes were detected in the vitreous cavity, with the linear echoes connected to the posterior wall near the optic disk (Figure 2E).

**Figure 3:**
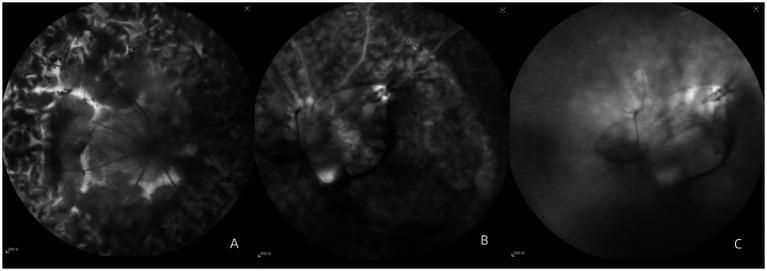
Fundus fluorescein angiography (FFA). **(A)** Late-phase FFA image of the right eye; **(B)** early-phase FFA image of the left eye; and **(C)** late-phase FFA image of the left eye.

Fundus fluorescein angiography (FFA) showed diffuse mixed hyperfluorescence and hypofluorescence in the right eye. The fibrotic areas exhibited hyperfluorescent staining without leakage, while pigmented hyperplasia areas showed hypofluorescence in the late phase (Figure 3A). In the left eye, early-phase FFA demonstrated masking fluorescence caused by vitreous opacities and hyperfluorescence due to neovascularization in the superior and inferior temporal regions of the optic disk. Arc-shaped hyperfluorescent staining was also observed in the temporal region (Figure 3B). In the late phase, optic disk leakage and diffuse retinal leakage were evident (Figure 3C).

Ancillary laboratory tests were performed to exclude infectious and autoimmune diseases. All systemic evaluations were unremarkable. The patient was ultimately diagnosed with bilateral diffuse SFU syndrome, VRT syndrome, and retinal neovascularization in the left eye. The right eye was considered to be in a stable stage; therefore, no treatment was administered. Given the presence of active uveitis, VRT syndrome, and retinal neovascular hemorrhage in the left eye, active treatment was initiated. Pars plana vitrectomy and intravitreal injection of triamcinolone acetonide were performed. Vitreous samples were collected intraoperatively for pathogen detection and inflammatory factor analysis. Diagnostic vitrectomy can not only relieve vitreoretinal traction and remove inflammatory factors from the vitreous but also allow for the collection of vitreous fluid for testing to help rule out infectious etiologies and masquerade syndromes, thereby guiding subsequent treatment. The intraocular fluid test results are shown in Table 1, revealing elevated levels of interleukin-6 (IL-6), IL-8, and vascular cell adhesion molecule (VCAM), a decreased IL-10/IL-6 ratio (<1.0), and negative pathogen testing.

**Table 1:** Medical laboratory test report.

No.	Test item	Result	Unit	Reference value	Method
1	VEGF	1.5	Pg/ml	0–160	Flow cytometry CBA
2	IL-6	104.1↑	Pg/ml	1–50	Flow cytometry CBA
3	IL-8	32.1↑	Pg/ml	0–20	Flow cytometry CBA
4	VCAM	7111.3↑	Pg/ml	200–1,000	Flow cytometry CBA
5	BFGF	0	Pg/ml	0–1	Flow cytometry CBA
6	IL-10	0.1	Pg/ml	0–5	Flow cytometry CBA
7	IL-10/IL-6	0.001			

CBA, cytometric bead array.

**Figure 4:**
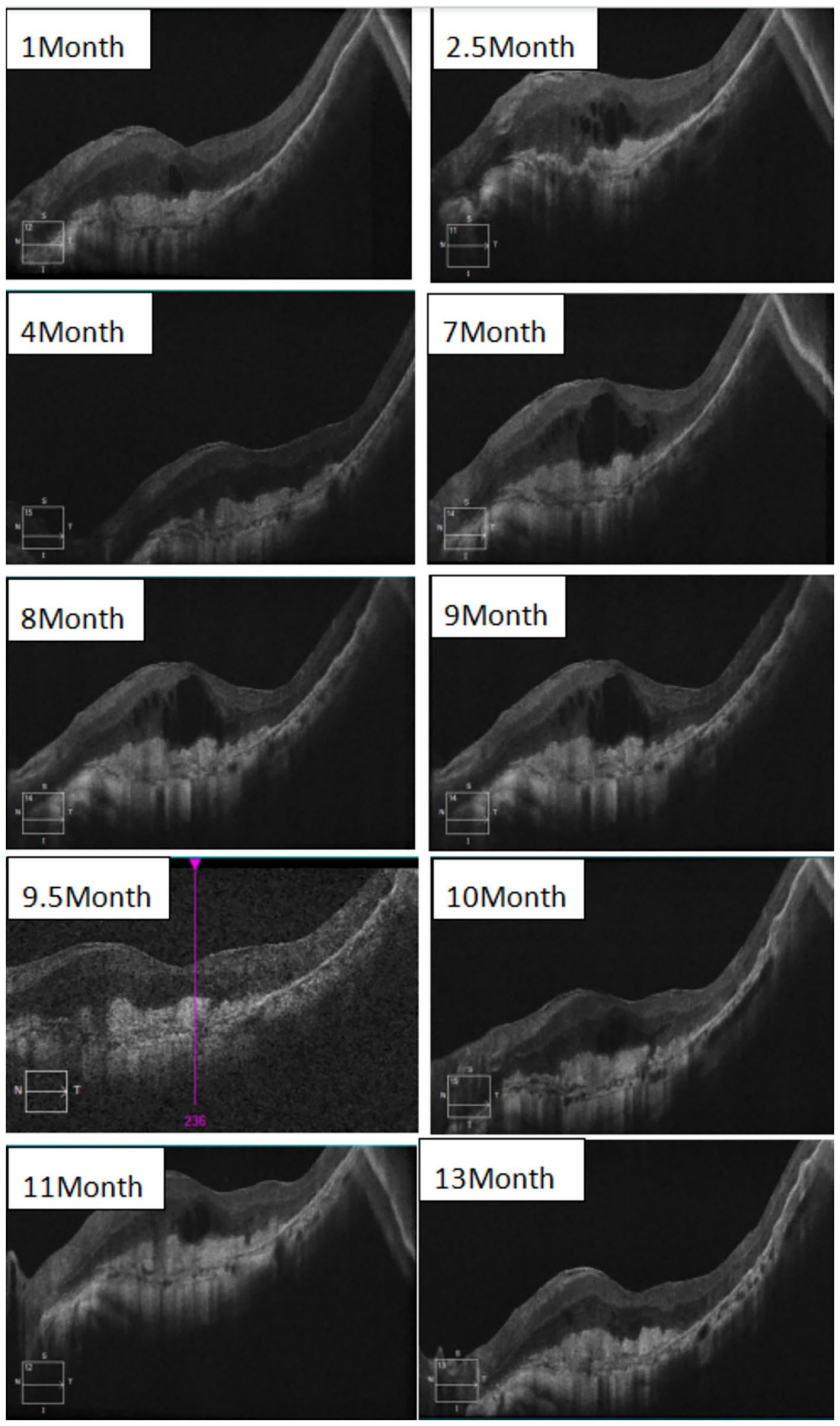
Postoperative macular OCT follow-up image of the left eye.

**Figure 5:**
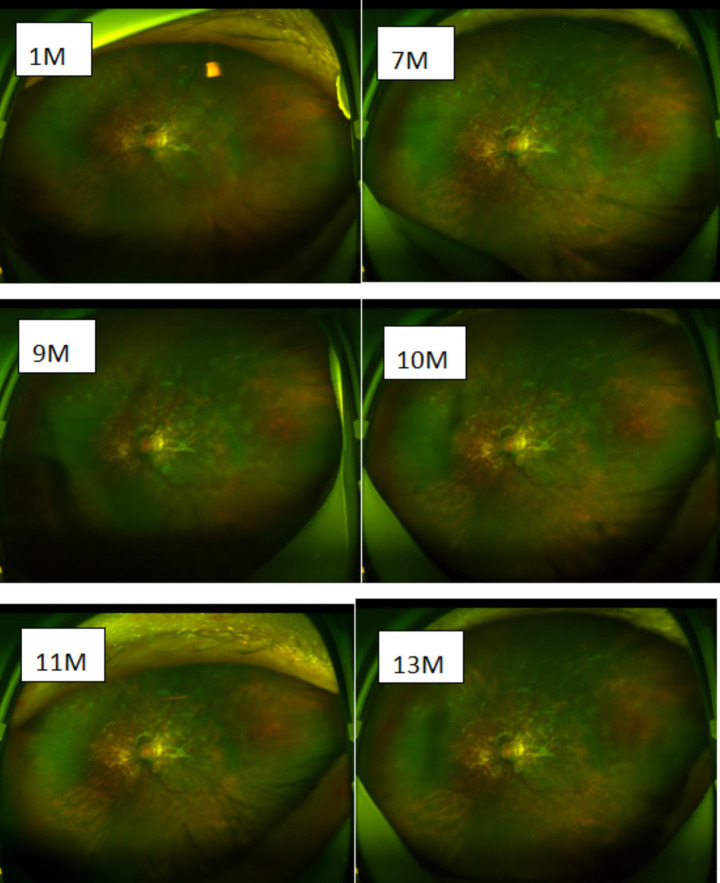
Postoperative follow-up macular fundus photograph of the left eye.

Considering that SFU is an immune-related inflammatory condition, postoperatively, oral glucocorticoid (prednisone) and the immunosuppressant ciclosporin were administered, along with gastric mucosal protection and potassium supplementation. Topical ocular treatment consisted of tobramycin/dexamethasone eye drops and diclofenac sodium eye drops for both eyes. The initial oral dose of prednisone was 30 mg, taken for 1 week, and then gradually tapered, and ciclosporin was given at 50 mg twice daily. However, on postoperative day 10, the patient reported significant gastrointestinal side effects that markedly affected appetite and rest, and requested a dose reduction. Consequently, the prednisone dose was reduced to 5 mg per day from day 10 onward, while the ciclosporin dose remained unchanged. During follow-up, the patient also experienced recurrent and worsening macular edema. Therefore, a posterior sub-Tenon injection of triamcinolone acetonide (20 mg) was administered, and the doses of prednisone and ciclosporin were adjusted accordingly. After discussing with the patient and their family, an intravitreal anti-vascular endothelial growth factor (VEGF) injection was administered after peribulbar triamcinolone for macular edema proved ineffective. Intravitreal injections of conbercept (0.5 mg) were given at 9 and 10 months postoperatively to manage macular edema, and achieved a certain degree of efficacy. At 11 months postoperatively, an intravitreal injection of faricimab (6 mg) was administered. A follow-up examination at 2 months showed no significant recurrence of macular edema. The postoperative treatment regimen and treatment outcomes are shown in Table 2 and Figures 4, 5.

**Table 2:** Postoperative treatment and outcomes.

Time point	Examination results	Treatment regimen
1 month	BCVA 0.05; no obvious inflammatory reaction in vitreous, retinal neovascularization regressed, subretinal fibrosis expanded, and macular edema alleviated.	Prednisone 5 mg once daily and ciclosporin 50 mg twice daily.
2.5 months	BCVA 0.05; recurrence of macular edema in the left eye.	Prednisone 5 mg once daily, ciclosporin 50 mg twice daily, and posterior sub-Tenon injection of 20 mg triamcinolone acetonide.
4 months	BCVA 0.05; macular edema resolved.	Prednisone 5 mg once daily and ciclosporin 50 mg twice daily.
7 months	BCVA 0.02; macular edema recurred, the degree of subretinal fibrosis increased compared to that at 1 month postoperatively.	Ciclosporin 50 mg once daily, and posterior sub-Tenon injection of 20 mg triamcinolone acetonide.
8 months	BCVA 0.02; macular edema.	Ciclosporin 50 mg once daily.
9 months	BCVA 0.02; persistent macular edema, but no significant progression of subretinal fibrosis.	Ciclosporin 50 mg once daily and intravitreal injection of conbercept.
9.5 months	BCVA 0.05; macular edema resolved.	Ciclosporin 50 mg once daily.
10 months	BCVA 0.05; mild macular edema.	Ciclosporin 50 mg once daily, intravitreal injection of conbercept.
11 months	BCVA 0.05; mild macular edema.	Ciclosporin 50 mg once daily, intravitreal injection of faricimab.
13 months	BCVA 0.05; macular edema further improved, and subretinal fibrosis remained stable with no significant progression.	Ciclosporin 50 mg once daily.

At the 1-month follow-up, visual acuity showed no significant improvement. The BCVA remained 0.05, although the patient reported slight subjective improvement. Fundus examination revealed resolution of vitreous inflammation and vitreoretinal traction, a clear optic disk margin, and the disappearance of neovascularization; however, the extent of subretinal fibrosis had increased. Macular OCT showed improved retinal layer definition and reduced edema, although residual cystoid edema and enlargement of the subretinal fibrotic area persisted. Based on Figure 5, the extent of subretinal fibrosis remained essentially stable after the 7th postoperative month, with no significant enlargement. However, macular edema recurred repeatedly.

## 3 Discussion

This study reports a rare case of bilateral diffuse SFU syndrome with VRT syndrome and retinal neovascularization in the left eye. The interval between the involvement of the two eyes exceeds 4 years. Most lesions in this condition are typically located in the posterior pole; however, the extent of lesions in this patient was unusually widespread. The coexistence of VRT syndrome and retinal neovascularization in the left eye has not been previously described in the literature.

The SFU syndrome was first described by Palestine in 1984 (2). Since then, only a limited number of cases have been reported, particularly those with diffuse bilateral involvement. The disease predominantly affects adults, although pediatric cases have also been described, with the youngest patients described at 3 years of age (13–15). The pathogenesis remains poorly understood. It has been suggested that SFU may be related to an autoimmune response involving the retinal pigment epithelium (RPE). The fibrotic component of SFU is believed to originate from RPE cells, and prolonged inflammation may lead to subretinal fibrous proliferation (2, 3), which contributes to severe visual impairment. Exuberant subretinal fibrous metaplasia is likely a non-specific response to severe tissue injury in this region. Immunohistopathological studies have demonstrated granulomatous lymphocytic infiltration in the choroid, predominantly driven by CD20-positive B cells with associated fibroblast activation. However, the underlying trigger remains unclear and may be related to infection or exposure to autoantigens (15–18).

Systemic diseases have been reported in association with SFU, including ectodermal dysplasia and ulcerative hemorrhagic colitis (19, 20). However, in this study, neither systemic evaluation nor intraocular fluid analysis identified any associated systemic condition. SFU is characterized by progressive subretinal fibrosis and may be accompanied by subretinal fluid, intraretinal fluid, outer retinal atrophy, macular stellate exudation, optic disk edema, and even choroidal neovascularization (CNV) (18, 21–23). To date, SFU complicated by VRT syndrome and retinal neovascularization has not been reported, and this case may represent the first such description.

Retinal neovascularization is a rare but serious complication of uveitis and may lead to vitreous hemorrhage, VRT, and even tractional retinal detachment, thereby posing a significant threat to vision. Inflammation is a key pathogenic mechanism. In this case, elevated levels of IL-6, IL-8, and VCAM were detected in the vitreous fluid, supporting the presence of inflammation. The differential diagnosis between SFU and masquerade syndromes, particularly PIOL, is critical, as both conditions can present with overlapping clinical features, including vitreous inflammation, subretinal infiltrates, and retinal pigment epithelium changes. Subretinal infiltrates in PIOL often appear as creamy or yellowish lesions beneath the retina, which may resemble the subretinal fibrosis seen in SFU. However, several key features aid differentiation. On multimodal imaging, OCT in PIOL typically reveals hyperreflective lesions at the level of the retinal pigment epithelium or subretinal space. In contrast, SFU is characterized by progressive subretinal fibrosis with less prominent cellular infiltration (24–26). In this case, the patient presented with significant vitreous proliferation and progressive subretinal fibrosis, with no obvious signs of cellular infiltration. Importantly, vitreous fluid testing played a significant role in supporting the differential diagnosis. PIOL is characterized by an elevated interleukin-10 (IL-10) level and an increased IL-10/IL-6 ratio (>1.0), whereas inflammatory conditions such as SFU typically show elevated IL-6 with a low IL-10/IL-6 ratio, as seen in this case. Although the cytopathologic examination of vitreous specimens remains the gold standard for diagnosing PIOL, combined with the patient’s multimodal imaging findings and vitreous testing essentially ruled out the diagnosis of lymphoma.

The treatment of SFU remains controversial and is not well standardized. There is currently no universally accepted therapeutic strategy. Given the presumed inflammatory basis of subretinal fibrosis, many investigators have used high-dose corticosteroids and immunosuppressive agents, such as cyclosporine (27) and cyclophosphamide (28). In recent years, tumor necrosis factor-*α* (TNF-α) monoclonal antibodies have also been explored, including infliximab and rituximab, a certain therapeutic effect was achieved, including improvement in visual acuity and inflammation control. Given that recent pathological findings suggest that B cells play an important role in the underlying pathogenesis of this disease, some experts believe that TNF-*α*, such as rituximab, should be considered a first-line therapy for this potentially devastating condition (23, 29). In this case, the plan was initially to use a combination of corticosteroids, immunosuppressants, and TNF-α inhibitors postoperatively. However, due to financial constraints, the patient and her family declined TNF-α inhibitor therapy, and the study opted for combination therapy with corticosteroids and immunosuppressants instead. For cases with macular edema, intravitreal ranibizumab has been attempted as an off-label rescue therapy. However, previous reports have indicated that ranibizumab did not provide functional or morphological improvement, and disease progression continued despite aggressive treatment with corticosteroids, immunosuppressants, and local therapy (30).

Although available treatments may stabilize the ocular structure by controlling active inflammation, visual acuity often does not improve. This is largely attributable to the formation of extensive fibrotic plaques at the posterior pole, resulting in substantial structural damage to the retina. In some cases, despite treatment with cyclophosphamide, infliximab, or rituximab, subretinal fibrosis continues to progress and coalesce, without stabilization or improvement in visual acuity, and may ultimately lead to irreversible visual loss. As shown in Figures 4, 5, in the patient, subretinal fibrosis remained largely stable after 7 months of maintenance therapy with immunosuppressants and low-dose corticosteroids, with no obvious enlargement or coalescence. However, macular edema recurred repeatedly. Ultimately, after discussion with the patient and her family, this study used anti-VEGF therapy off-label to treat macular edema and achieved some therapeutic effect. When the patient had significant macular edema, such as at 7 months postoperatively, she reported decreased visual acuity (0.02); after the macular edema resolved, her visual acuity improved (0.05). However, due to the formation of outer retinal plaques, her visual acuity could not improve further, and the best-corrected visual acuity remained at approximately 0.05. Overall, SFU syndrome is associated with a poor prognosis, and it is important to inform patients accordingly during treatment.

Both eyes may be affected either simultaneously or sequentially, with potentially long intervals between involvement. In the present case, the interval exceeded 4 years. Subretinal fibrosis had already involved the macular region at presentation. Although VRT was relieved, retinal neovascularization regressed, intraretinal edema was reduced, and inflammation was partially controlled after treatment, visual acuity did not improve significantly, and subretinal fibrosis continued to progress. In this case, after more than 6 months of active treatment, subretinal fibrosis can be controlled to a certain extent. However, the recurrent nature of macular edema remains a treatment challenge. The risk of further fibrosis, macular edema, or secondary CNV in the left eye remains a persistent clinical concern.

In summary, this study reports a rare case of diffuse SFU syndrome complicated by VRT syndrome. This case highlights that SFU may develop with a prolonged interocular interval and may be associated with retinal neovascularization and VRT syndrome. Close attention should be paid to the progression of subretinal fibrosis, recurrent macular edema, and the potential development of CNV during treatment.

## Data Availability

The original contributions presented in the study are included in the article/supplementary material; further inquiries can be directed to the corresponding author.
